# Factors associated with large watery stools after out-of-hospital cardiac arrest and their relationship with neurological outcomes: A retrospective observational study

**DOI:** 10.1016/j.resplu.2025.100946

**Published:** 2025-03-26

**Authors:** Yasuyuki Kawai, Keita Miyazaki, Toru Osaki, Koji Yamamoto, Keisuke Tsuruta, Hideki Asai, Hidetada Fukushima

**Affiliations:** Department of Emergency and Critical Care Medicine, Nara Medical University, 840 Shijo-cho, Kashihara, Nara 634-8522, Japan

**Keywords:** Cardiopulmonary arrest, Watery stool, Non-occlusive mesenteric ischaemia, Mesenteric ischaemia

## Abstract

**Aim:**

To evaluate the association between large watery stools—a potential early sign of non-occlusive mesenteric ischaemia—and neurological outcomes.

**Methods:**

We retrospectively analysed data from 495 adult patients with out-of-hospital cardiac arrest admitted to our tertiary centre between April 2015 and March 2024. Individuals who achieved return of spontaneous circulation but remained comatose after resuscitation were included. Large watery stools were defined as ≥300 mL of watery/loose stools occurring at least twice within 24 h after admission. We performed stepwise logistic regression analysis to identify predictors available at admission and assess the relationship between watery stool occurrence and neurological outcomes.

**Results:**

Overall, 161 (32%) patients developed large watery stools within 24 h. Patients with watery stools experienced significantly higher rates of unfavourable neurological outcomes at discharge (favourable in 9% vs. 21% in patients without watery stools). Stepwise logistic regression analysis identified six independent predictors of large watery stools—lactate, low-flow interval, C-reactive protein, activated partial thromboplastin time, noradrenaline use, and creatinine—reflecting disturbances in metabolic status, perfusion, coagulation, and inflammatory responses. The final model demonstrated an ROC_AUC of 0.72 (95% CI [0.68–0.73]). After calibration, the Brier score improved from 0.21 (95% CI [0.20–0.23]) to 0.19 (95% CI [0.17–0.21]).

**Conclusion:**

After out-of-hospital cardiac arrest, early-onset large watery stools is strongly associated with poor neurological outcomes, potentially serving as a clinical indicator of intestinal ischaemia and systemic inflammation. Incorporating coagulation and metabolic markers into predictive models may facilitate early identification of high-risk patients, aiding timely diagnosis and intervention.

## Highlights

• Early watery stools predict poor outcomes in survivors of OHCA.
• Admission activated partial thromboplastin time predicts risk of watery stools.
• Early prediction of watery stools may guide interventions for bowel ischaemia.

## Introduction

Patients with out-of-hospital cardiac arrest (OHCA) often have extremely poor neurological outcomes, even after successful resuscitation and admission to the intensive care unit (ICU).^1^ One factor underlying these poor outcomes is post-cardiac arrest syndrome, which involves global organ dysfunction induced by ischaemia–reperfusion injury.^2^, ^3^

The pathophysiology of post-cardiac arrest syndrome includes non-occlusive mesenteric ischaemia (NOMI),^4^ which is characterised by mucosal ischaemia and necrosis due to intestinal hypoperfusion. NOMI can lead to intestinal ischaemia with necrosis. Moreover, if not diagnosed and treated promptly, NOMI can result in severe post-resuscitation complications, including multi-organ failure and death.^5^, ^6^ However, during the immediate post-resuscitation period, clinical assessment of the abdomen—such as pain elicitation—may be challenging due to impaired neurological function and sedation, delaying the diagnosis of NOMI.^7^

Recent observations suggest that large watery stools, often noted during the post-resuscitation period, may constitute an early sign of bowel mucosal injury or ischaemia.^8^ However, the relationship between this clinical sign, NOMI, and patient outcomes has not been fully clarified.

In this study, we aimed to evaluate the association between large watery stools—identified as a potential early sign of NOMI—and neurological outcomes. The study also sought to determine whether resuscitation-related factors identifiable at hospital arrival could predict the onset of large watery stools at an early stage. Early recognition and appropriate intervention for NOMI may help mitigate the progression of systemic inflammatory responses and multi-organ failure, thereby improving neurological outcomes. The results of this study may enhance the diagnostic accuracy for intestinal ischaemia and prompt a re-evaluation of current treatment strategies for OHCA.

## Materials and methods

### Study design

This study was approved by the Ethics Committee of Nara Medical University (approval number: 3916). Because this was a retrospective observational study, an opt-out approach to consent was used. All data were anonymised. The study adhered to the tenets of the Declaration of Helsinki.

### Study population and data source

The study included patients aged ≥18 years who were admitted to our institution for post-resuscitation management after OHCA between April 2015 and March 2024. All data were collected continuously. No sample size calculation or power analysis was performed in this study. Instead, we aimed to collect the maximum amount of information available during the observation period to ensure comprehensive data analysis, capture a wide range of real-world clinical scenarios, and increase the robustness of our findings. The exclusion criteria were as follows: (1) non-comatose patients, (2) patients who underwent abdominal surgery or interventional radiology after resuscitation (not for NOMI), (3) patients with known inflammatory bowel disease or pre-existing gastrointestinal symptoms, (4) patients with intra-abdominal or gastrointestinal bleeding, and (5) patients who died within 6 h of ICU admission. Data were obtained from our critical and acute care patient information systems (PIMS: 2015–2016, ACSYS: 2017–2024; both manufactured by Philips, Netherlands) and electronic medical records (2015–2024). PIMS and ACSYS routinely record patient conditions every 2 h from the time of admission. In addition, these systems document stool frequency, characteristics, and weight (g) from arrival and throughout the hospital stay; if the amount was too large to measure, it was documented accordingly.

### Data collection and features

The following data were collected: patient background, Utstein-style resuscitation record,^9^ blood test results obtained upon hospital arrival, stool information, and neurological outcomes at discharge. For this study, “return of spontaneous circulation (ROSC)” refers to the patient’s status upon ICU admission. All patients initially achieved ROSC; however, those who did not maintain sustained circulation upon ICU arrival were managed with extracorporeal membrane oxygenation (ECMO). Neurological outcomes were categorised using the Cerebral Performance Category (CPC),^10^ with CPC 1–2 defined as favourable and CPC 3–5 as unfavourable. Large watery stools were defined as ≥300 mL of watery or loose stools occurring at least twice within 24 h of resuscitation.^8^ There are no clear volume criteria to define watery stools; however, the 300 mL standard—widely used in intensive care—was used in this study.^11^, ^12^ The data collection window spanned from hospital arrival to ICU admission. However, in our clinical practice, large watery stools were observed and documented exclusively after ICU admission, with no cases observed and recorded before ICU entry.

### Features and data preprocessing

Features identifiable prior to ICU admission were selected based on a previous study,^4^ as detailed in Supplement 1. Categorical variables were converted to numerical values using one-hot encoding, and continuous variables were standardised. Missing values were imputed using multiple imputation (random forest) (eMethods 1 and 2 in Supplement 2), considering the correlations among variables (Supplement 3).

### Statistical analysis and logistic regression

First, we examined the normality of continuous variables using the Shapiro–Wilk test. Because most variables did not follow a normal distribution, continuous variables are expressed as median (interquartile range), while categorical variables are expressed as n (%). Between-group differences were assessed using the Mann–Whitney test or Fisher’s exact test. All statistical tests were two-tailed, and a *p*-value of <0.05 was considered statistically significant.

Next, we performed a logistic regression analysis to explore factors associated with the occurrence of large watery stools. Variable selection was conducted using a stepwise method (combining forward selection and backward elimination) based on *p*-values; only variables that met the inclusion criteria (*p* < 0.05) and remained significant (*p* < 0.10 for removal) were retained in the final model. The final model was evaluated using the area under the receiver operating characteristic (ROC) curve (ROC_AUC), confusion matrix, sensitivity, specificity, precision, recall, and F1 score. In addition, CalibratedClassifierCV (via isotonic method) (eMethod 3 in Supplement 2) was used to correct predicted probabilities, comparing the Brier score and other metrics before and after calibration. Finally, we identified the threshold that maximised the F1 score**.**

In addition to our primary analysis, we performed a supplementary multivariable logistic regression analysis to examine if watery stools at admission, along with other clinical variables identified upon admission, could predict neurological outcomes (CPC 1–2). The variable ‘watery stools’ was incorporated into the model, along with other early identifiable clinical variables.

All statistical analyses and machine learning procedures were performed using Python 3.10.12 (Python Software Foundation, Wilmington, DE, USA).

## Results

### Patient background and occurrence of large watery stools

**Fig. 1:**
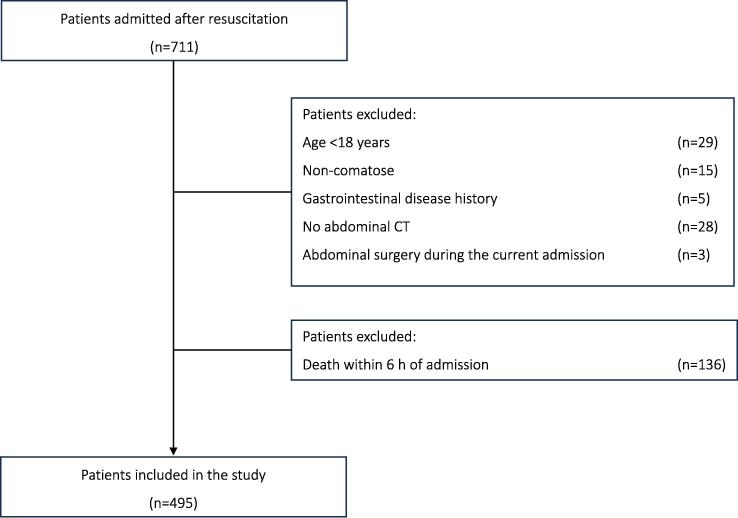
Patient selection flow.

During the study period, 711 patients were admitted for post-resuscitation management of OHCA, and 495 were included in the analysis after the exclusion criteria were applied (Fig. 1). Notably, although the retrospective observational nature of our study precluded definitive assessment, none of these cases underwent laparotomy or angiographic procedures for suspected NOMI based on the available records. Of these patients, 161 (32%) developed large watery stools within the first 24 h of admission. Patients in this group had significantly longer low-flow intervals and higher ECMO usage than those without diarrhoea (both *p* < 0.001). Metabolic abnormalities were more pronounced, with higher lactate levels and lower arterial pH observed in the diarrhoea group (both *p* < 0.001). Coagulation abnormalities were also notable, with a prolonged activated partial thromboplastin time (APTT) and elevated D-dimer levels in patients in the diarrhoea group (both *p* < 0.001). Neurological outcomes were significantly worse in the diarrhoea group, with only 9% achieving a favourable outcome (CPC 1/2) compared with 21% in the group without diarrhoea (*p* = 0.001). These findings suggest a strong association between the development of large watery stools and markers of metabolic stress, coagulation abnormalities, and poor neurological outcomes (Table 1).

**Table 1:** Baseline characteristics of patients with out-of-hospital cardiac arrest (OHCA) with and without large watery stools.

		Total cases	Diarrhoea	Non-diarrhoea	*p*-value
		*n* = 495	*n* = 161	*n* = 334	
Age (years), median (IQR)		70 (58–79)	69 (56–79)	71 (59–79)	0.2
Sex (male), *n* (%)		322 (65)	105 (65)	217 (65)	>0.99
Risk factors, *n* (%)	Diabetes	85 (17)	25 (16)	60 (18)	0.5
	Anticoagulant use	74 (15)	17 (11)	57 (17)	0.06
	Cancer	21 (4)	7 (4)	14 (4)	1
	Haemodialysis	16 (3)	4 (3)	12 (4)	0.6
Witness, *n* (%)		343 (69)	103 (64)	240 (72)	0.08
Bystander CPR, *n* (%)		224 (45)	74 (46)	140 (45)	0.8
Shockable rhythm, *n* (%)		114 (23)	34 (21)	80 (24)	0.6
With physician		245 (50)	67 (42)	178 (53)	0.02
Adrenaline dose (mg)		2 (1–3)	2 (1–4)	1 (0–3)	<0.001
Defibrillation performed, *n* (%)		138 (28)	55 (34)	83 (25)	0.03
No-flow interval (min), median (IQR)		0 (0, 9)	4 (0, 9)	0 (0, 9)	0.1
Low-flow interval (min), median (IQR)		32 (18–50)	41 (25–57)	28 (15–44)	<0.001
ROSC, *n* (%)		453 (92)	138 (86)	315 (94)	0.002
ECMO used, *n* (%)		89 (18)	45 (28)	44 (13)	<0.001
Impella used, *n* (%)		12 (2)	6 (4)	6 (2)	0.2
TTM performed, *n* (%)		224 (49)	69 (43)	175 (53)	0.045
Right pupil diameter (mm), median (IQR)		4.0 (2.9–5.3)	4.6 (3.3–5.6)	3.8 (2.7–5.0)	<0.001
Left pupil diameter (mm), median (IQR)		4.0 (2.9–5.2)	4.5 (3.5–5.7)	3.7 (2.7,5.0)	<0.001
Blood gas, median (IQR)	pH	6.95 (6.83–7.09)	6.88 (6.77–7.01)	6.99 (6.85–7.15)	<0.001
	pO_2_ (mmHg)	106 (59–243)	83 (51–202)	110 (63–251)	0.043
	pCO_2_ (mmHg)	71 (47–96)	77 (55–101)	69 (45–92)	0.005
	Lactate (mmol/L)	11.7 (8.5–15.0)	13.9 (10.6–17.0)	10.7 (7.9–14.0)	<0.001
Blood test, median (IQR)	White blood cells (×10^3^/µl)	10.4 (7.7–13.4)	10.8 (7.6–13.4)	10.2 (7.7–13.5)	0.7
	Haemoglobin (g/L)	11.8 (10.2–13.6)	11.8 (10.1–13.7)	11.8 (10.3–13.5)	0.8
	C-reactive protein (mg/dL)	0.2 (0.0–1.8)	0.1 (0.0–1.4)	0.2 (0.15–2.1)	0.2
	Albumin (g/dL)	3.3 (2.9–3.7)	3.3 (3.0–3.7)	3.3 (2.9–3.7)	0.9
	AST (U/L)	112 (57–222)	151 (69–305)	98 (55–196)	<0.001
	ALT (U/L)	74 (35–161)	107 (46–211)	63 (31–145)	<0.001
	LDH (U/L)	422 (324–622)	498 (358–759)	398 (313–572)	<0.001
	Cre (mg/dL)	1.11 (0.86–1.43)	1.16 (0.95–1.44)	1.08 (0.83–1.39)	0.02
	PT (s)	15.3 (13.7–17.8)	16.0 (14.5–17.8)	15.0 (13.4–17.8)	0.002
	INR	1.26 (1.14–1.48)	1.31 (1.20–1.45)	1.22 (1.11–1.48)	<0.001
	APTT (s)	37.9 (30.5–49.0)	43.0 (35.3–57.7)	34.8 (28.5–44.2)	<0.001
	Fibrinogen (mg/dL)	281 (222–367)	266 (204–338)	293 (235–384)	0.001
	FDP (mg/L)	37.5 (16.9–80.9)	47.8 (24.8–109.0)	32.4 (14.3–68.5)	<0.001
	D-dimer (ng/mL)	19.7 (8.3–38.1)	24.6 (12.4–50.5)	15.9 (7.1–32.5)	<0.001
Vasopressor used upon ICU admission, *n* (%)	Adrenaline	72 (15)	26 (16)	46 (14)	0.02
	Noradrenaline	172 (35)	67 (42)	105 (31)	
	Dobutamine	9 (2)	4 (3)	5 (2)	
	Dopamine	12 (2)	1 (1)	11 (3)	
Survival to discharge, *n* (%)		167 (34)	35 (22)	132 (40)	<0.001
CPC 1/2, *n* (%)		83 (17)	14 (9)	69 (21)	0.001

IQR, interquartile range; CPR, cardiopulmonary resuscitation; ROSC, return of spontaneous circulation; ECMO, extracorporeal membrane oxygenation; TTM, targeted temperature management; CPC, Cerebral Performance Category; ICU, intensive care unit; ALT, alanine aminotransferase, APTT, activated partial thromboplastin time; AST, aspartate aminotransferase; FDP, fibrin/fibrinogen degradation products; LDH, lactate dehydrogenase; PT, prothrombin time.

### Factor selection and model performance using logistic regression analysis

**Fig. 2:**
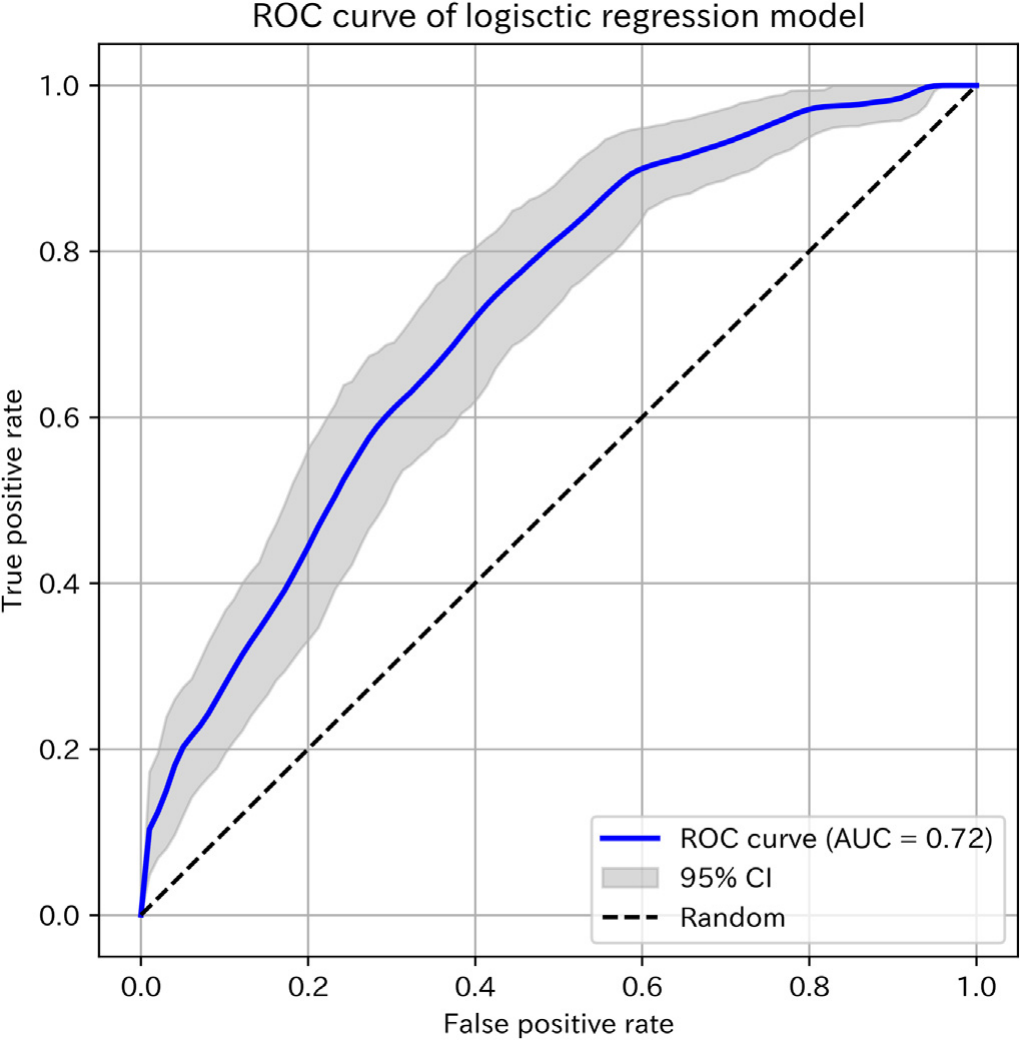
**Receiver operating characteristic curves for the logistic regression model.** Receiver operating characteristic curves with 95% confidence intervals for the logistic regression model (AUC = 0.72). The dashed line represents the performance of a random classifier. AUC, area under the curve; CI, confidence interval; ROC, receiver operating characteristic; TPR, true positive rate; FPR, false positive rate.

Stepwise logistic regression analysis identified six variables—lactate, low-flow interval, C-reactive protein, APTT, noradrenaline use, and creatinine—as independent predictors of large watery stools within the first 24 h after admission (Table 2). The model converged after 18 iterations (pseudo *R*^2^ = 0.12, LLR *p*-value < 0.001) and demonstrated a ROC_AUC of 0.72 (95% confidence interval (CI) [0.68–0.73]) (Fig. 2). After calibration, the Brier score improved from 0.21 (95% CI [0.20–0.23]) to 0.19 (95% CI [0.17–0.21]) (Supplement 4), with moderate sensitivity and specificity (Table 3).

**Table 2:** Logistic regression analysis of factors associated with large watery stools within 24 h post-OHCA.

Variables	Odds ratio	VIF	95% confidence intervals	*p*-value
Intercept	0.43	11.04	(−1.07, −0.64)	<0.001
Lactate (mmol/L)	1.63	1.32	(0.25, 0.73)	<0.001
Low-flow interval (min)	1.39	1.20	(0.11, 0.55)	0.003
C-reactive protein (mg/dL)	0.66	1.08	(−0.67, −0.16)	0.001
APTT (s)	1.37	1.25	(0.09, 0.54)	0.006
Noradrenaline use	1.31	1.01	(0.07, 0.47)	0.01
Creatinine (mg/dL)	1.25	1.04	(0.02, 0.42)	0.03

Pseudo *R*^2^, 0.12; LLR *p*-value, 1.60e−14.

This table presents odds ratios, 95% confidence intervals, and *p*-values from the final stepwise logistic regression model.

**Table 3:** Confusion matrices and key metrics for the logistic regression model.

Confusion matrix of the logistic regression model			
Actual	Predicted: No diarrhoea	Predicted: Diarrhoea	Metrics
No diarrhoea	145	189	Specificity: 0.43
Diarrhoea	18	143	Sensitivity: 0.89
	NPV: 0.89	PPV: 0.43	

NPV, negative predictive value; PPV, positive predictive value.

In the additional analysis predicting favourable neurological outcomes (CPC 1–2) (Supplement 5), the variable ‘watery stools’ was incorporated into the model with other early identifiable clinical variables. While watery stools did not identified as an independent predictor, other early markers such as elevated lactate, prolonged low-flow interval, and prolonged APTT were significantly associated with CPC outcomes. These findings suggest that watery stools may indicate similar underlying physiological disturbances, consistent with our primary analysis (Table 2).

## Discussion

A higher proportion of patients in this study developed large watery stools within 24 h of OHCA onset than in a study by Schriefl et al.^8^ These patients also exhibited significantly poorer neurological outcomes. Stepwise logistic regression analysis identified six independent predictors of large watery stools, indicating disruptions in metabolic status, perfusion, coagulation, and inflammatory responses. Notably, few studies have specifically focused on large watery stools as an early clinical sign, making this investigation one of the first large-scale analyses to demonstrate a clear association between watery stools and neurological outcomes after OHCA.

These findings highlight that the predictive performance achieved by our stepwise logistic regression model is not merely a statistical improvement but has tangible clinical implications. Early identification of patients at high risk of developing large watery stools can prompt clinicians to pursue prompt diagnostic imaging and initiate early therapeutic measures, ultimately contributing to improved patient outcomes.

In the additional analysis, watery stools did not independently predict neurological outcomes. This is likely because of the strong correlation of watery stools with other admission-related markers of severe systemic derangement, such as elevated lactate levels, prolonged low-flow interval, and elevated APTT (as demonstrated in our primary analysis, Table 2). While watery stools did not emerge as an independent predictor, it remains clinically relevant as part of a broader set of early indicators of systemic injury, consistent with real-world clinical practice.

Diagnosing NOMI remains challenging because of a lack of early, specific clinical signs.^6^ Although contrast-enhanced CT and endoscopy can provide diagnostic information,^7^, ^13^ their invasive nature and high resource demands limit routine use in all patients with suspected intestinal ischaemia. In this context, our study highlights large watery stools as a relatively common yet understudied clinical sign, suggesting its potential role as an early indicator of intestinal ischaemia. While the presence of watery stools alone does not confirm NOMI, early recognition of this symptom can prompt clinicians to prioritise further diagnostic imaging and initiate timely therapeutic interventions. This approach may help prevent systemic inflammatory responses, multi-organ failure, and secondary neurological deterioration, ultimately improving patient outcomes.

In this study, APTT emerged as a particularly strong predictor of large watery stools in the early post-resuscitation period. Because APTT primarily reflects the intrinsic and common coagulation pathways, it may more effectively capture acute coagulation disturbances induced by ischaemia–reperfusion injury and excessive fibrinolysis. In the context of post-resuscitation intestinal ischaemia, mucosal injury and barrier disruption can further amplify inflammatory and coagulation cascades. These findings suggest that APTT may serve as a valuable early indicator of intestinal ischaemia and help identify patients at risk of complications such as NOMI.

Although large watery stools were strongly associated with poor neurological outcomes, a small number of patients in our cohort achieved favourable outcomes despite developing this symptom. Therefore, clinicians should regard large watery stools not as an absolute prognostic determinant but as a sentinel event that signals the need for close monitoring and aggressive management, including circulatory optimisation, potential endovascular treatment, and anticoagulation therapy. Early and appropriate interventions could not only reduce NOMI-related mortality but also improve systemic and neurological recovery.

### Limitations

This was a single-centre, retrospective, observational study, limiting its external validity. To generalise model performance, verification through multicentre or international collaborative research is essential. In addition, large watery stools can result from various conditions other than intestinal ischaemia, including drug-induced diarrhoea, infection,^14^ and enteral feeding.^15^ Consequently, confirmation of NOMI requires additional diagnostic imaging, endoscopic procedures, or angiography. Furthermore, our model showed only moderate accuracy; thus, refinement is needed for broader clinical application. Since the primary aim was to identify high-risk groups using features available at hospital admission, incorporating additional data known to aid in the diagnosis of NOMI—such as abdominal CT findings—could improve predictive accuracy. Another limitation of our study is the temporal ambiguity inherent in the data collection process. Although data were chronologically collected from hospital arrival until ICU admission, watery stools were only documented after ICU admission. This limitation prevents us from determining the exact onset time of watery stools relative to the early admission variables. Future prospective studies should use a more precise, time-specific data collection protocol to clarify these temporal relationships.

### Future perspectives

To establish effective early diagnosis and treatment strategies for NOMI, the integration of continuous haemodynamic parameters, contrast-enhanced CT findings,^6^ biomarkers,^16^, ^17^ and angiography data into predictive models is warranted. Moreover, combining these approaches with high-precision models predicting post-resuscitation neurological outcomes may help identify patients at risk of intestinal ischaemia who otherwise may experience favourable neurological recovery. Prospective multicentre studies are essential for developing and validating these prediction tools.

## Conclusions

This study demonstrated that early-onset large watery stools in patients with OHCA are strongly associated with unfavourable neurological outcomes, potentially indicating underlying intestinal ischaemia. Integrating coagulation and metabolic markers into predictive models may enable earlier recognition of at-risk patients, prompting timely diagnostic imaging and targeted interventions. Such measures could mitigate NOMI-related complications and improve neurological recovery. However, these findings warrant further prospective validation through larger, multicentre studies.

## CRediT authorship contribution statement

**Yasuyuki Kawai:** Writing – review & editing, Writing – original draft, Visualization, Validation, Software, Methodology, Investigation, Formal analysis, Conceptualization. **Keita Miyazaki:** Writing – review & editing, Validation, Investigation, Conceptualization. **Toru Osaki:** Writing – review & editing, Validation, Investigation, Conceptualization. **Koji Yamamoto:** Writing – review & editing, Validation, Methodology, Investigation. **Keisuke Tsuruta:** Writing – review & editing, Validation, Methodology, Investigation. **Hideki Asai:** Writing – review & editing. **Hidetada Fukushima:** Writing – review & editing.

## Funding

This research did not receive any specific grant from funding agencies in the public, commercial, or not-for-profit sectors.

## Data availability

The datasets generated and/or analysed during the current study are available from the corresponding author upon reasonable request.

## Declaration of competing interest

The authors declare that they have no known competing financial interests or personal relationships that could have appeared to influence the work reported in this paper.
