# Supplementary material for: Factors associated with large watery stools after out-of-hospital cardiac arrest and their relationship with neurological outcomes: A retrospective observational study
Source: Resusc Plus. 2025 Mar 26;23:100946. doi: 10.1016/j.resplu.2025.100946 (PMC12002823; doi:10.1016/j.resplu.2025.100946)
Supplement: Supplementary Data 1 [file mmc1.pptx]

## Slide 1
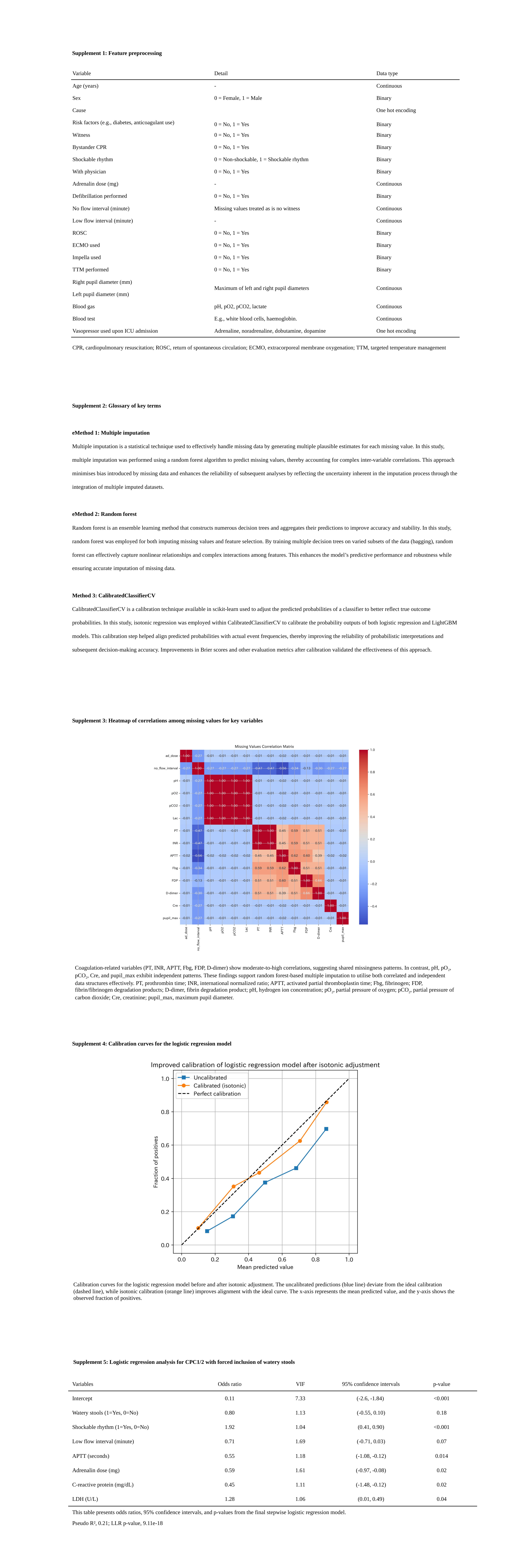

Supplement 1: Feature preprocessing
| Variable | Detail | Data type |
| --- | --- | --- |
| Age (years) | - | Continuous |
| Sex | 0 = Female, 1 = Male | Binary |
| Cause | | One hot encoding |
| Risk factors (e.g., diabetes, anticoagulant use) | 0 = No, 1 = Yes | Binary |
| Witness | 0 = No, 1 = Yes | Binary |
| Bystander CPR | 0 = No, 1 = Yes | Binary |
| Shockable rhythm | 0 = Non-shockable, 1 = Shockable rhythm | Binary |
| With physician | 0 = No, 1 = Yes | Binary |
| Adrenalin dose (mg) | - | Continuous |
| Defibrillation performed | 0 = No, 1 = Yes | Binary |
| No flow interval (minute) | Missing values treated as is no witness | Continuous |
| Low flow interval (minute) | - | Continuous |
| ROSC | 0 = No, 1 = Yes | Binary |
| ECMO used | 0 = No, 1 = Yes | Binary |
| Impella used | 0 = No, 1 = Yes | Binary |
| TTM performed | 0 = No, 1 = Yes | Binary |
| Right pupil diameter (mm) | Maximum of left and right pupil diameters | Continuous |
| Left pupil diameter (mm) | | |
| Blood gas | pH, pO2, pCO2, lactate | Continuous |
| Blood test | E.g., white blood cells, haemoglobin. | Continuous |
| Vasopressor used upon ICU admission | Adrenaline, noradrenaline, dobutamine, dopamine | One hot encoding |
| CPR, cardiopulmonary resuscitation; ROSC, return of spontaneous circulation; ECMO, extracorporeal membrane oxygenation; TTM, targeted temperature management | | |
Supplement 2: Glossary of key terms
eMethod 1: Multiple imputation
Multiple imputation is a statistical technique used to effectively handle missing data by generating multiple plausible estimates for each missing value. In this study, multiple imputation was performed using a random forest algorithm to predict missing values, thereby accounting for complex inter-variable correlations. This approach minimises bias introduced by missing data and enhances the reliability of subsequent analyses by reflecting the uncertainty inherent in the imputation process through the integration of multiple imputed datasets.
eMethod 2: Random forest
Random forest is an ensemble learning method that constructs numerous decision trees and aggregates their predictions to improve accuracy and stability. In this study, random forest was employed for both imputing missing values and feature selection. By training multiple decision trees on varied subsets of the data (bagging), random forest can effectively capture nonlinear relationships and complex interactions among features. This enhances the model’s predictive performance and robustness while ensuring accurate imputation of missing data.
Method 3: CalibratedClassifierCV
CalibratedClassifierCV is a calibration technique available in scikit-learn used to adjust the predicted probabilities of a classifier to better reflect true outcome probabilities. In this study, isotonic regression was employed within CalibratedClassifierCV to calibrate the probability outputs of both logistic regression and LightGBM models. This calibration step helped align predicted probabilities with actual event frequencies, thereby improving the reliability of probabilistic interpretations and subsequent decision-making accuracy. Improvements in Brier scores and other evaluation metrics after calibration validated the effectiveness of this approach.
Supplement 3: Heatmap of correlations among missing values for key variables
Coagulation-related variables (PT, INR, APTT, Fbg, FDP, D-dimer) show moderate-to-high correlations, suggesting shared missingness patterns. In contrast, pH, pO2, pCO2, Cre, and pupil_max exhibit independent patterns. These findings support random forest-based multiple imputation to utilise both correlated and independent data structures effectively. PT, prothrombin time; INR, international normalized ratio; APTT, activated partial thromboplastin time; Fbg, fibrinogen; FDP, fibrin/fibrinogen degradation products; D-dimer, fibrin degradation product; pH, hydrogen ion concentration; pO2, partial pressure of oxygen; pCO2, partial pressure of carbon dioxide; Cre, creatinine; pupil_max, maximum pupil diameter.
Supplement 4: Calibration curves for the logistic regression model
Calibration curves for the logistic regression model before and after isotonic adjustment. The uncalibrated predictions (blue line) deviate from the ideal calibration (dashed line), while isotonic calibration (orange line) improves alignment with the ideal curve. The x-axis represents the mean predicted value, and the y-axis shows the observed fraction of positives.
Supplement 5: Logistic regression analysis for CPC1/2 with forced inclusion of watery stools
| Variables | Odds ratio | VIF | 95% confidence intervals | p-value |
| --- | --- | --- | --- | --- |
| Intercept | 0.11 | 7.33 | (-2.6, -1.84) | <0.001 |
| Watery stools (1=Yes, 0=No) | 0.80 | 1.13 | (-0.55, 0.10) | 0.18 |
| Shockable rhythm (1=Yes, 0=No) | 1.92 | 1.04 | (0.41, 0.90) | <0.001 |
| Low flow interval (minute) | 0.71 | 1.69 | (-0.71, 0.03) | 0.07 |
| APTT (seconds) | 0.55 | 1.18 | (-1.08, -0.12) | 0.014 |
| Adrenalin dose (mg) | 0.59 | 1.61 | (-0.97, -0.08) | 0.02 |
| C-reactive protein (mg/dL) | 0.45 | 1.11 | (-1.48, -0.12) | 0.02 |
| LDH (U/L) | 1.28 | 1.06 | (0.01, 0.49) | 0.04 |
| This table presents odds ratios, 95% confidence intervals, and p-values from the final stepwise logistic regression model. Pseudo R², 0.21; LLR p-value, 9.11e-18 | | | | |
